# Role of Cholesterol in Interaction of Ionic Liquids with Model Lipid Membranes and Associated Permeability

**DOI:** 10.1021/acs.jpcb.4c01531

**Published:** 2024-05-25

**Authors:** Sandeep Kumar, Navleen Kaur, Prashant Hitaishi, Sajal Kumar Ghosh, Venus Singh Mithu, Holger A. Scheidt

**Affiliations:** † Department of Chemistry, Guru Nanak Dev University, Amritsar 143005, India; ‡ Department of Physics, School of Natural Sciences, Shiv Nadar Institute of Eminence, NH91, Tehsil Dadri, G. B. Nagar, Greater Noida 201314, Uttar Pradesh, India; § Institute for Medical Physics and Biophysics, Leipzig University, Leipzig 04107, Germany

## Abstract

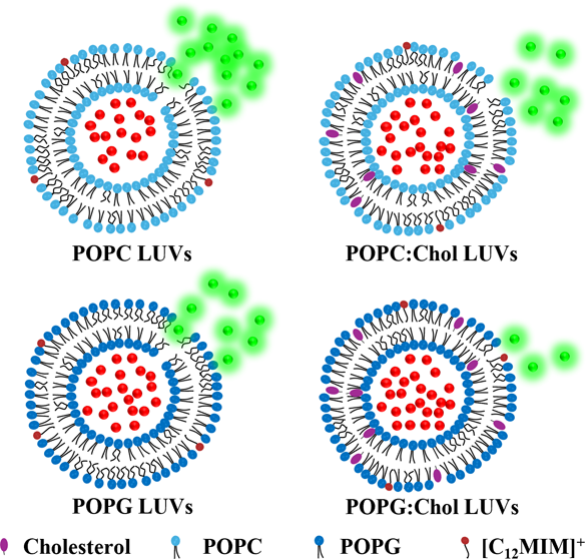

In this work, we explored how the amount of cholesterol in the lipid membrane composed of phosphatidylcholine (POPC) or phosphatidylglycerol (POPG) affects the interaction with 1-dodecyl-3-methylimidazolium bromide ([C_12_MIM]^+^Br^–^) ionic liquids using various biophysical techniques. On interacting with the membrane, [C_12_MIM]^+^Br^–^ leads to enhanced membrane permeability and induces membrane fusion, leading to an increase in vesicle size. The ^2^H-based solid-state NMR investigations of cholesterol-containing lipid membranes reveal that [C_12_MIM]^+^Br^–^ decreases the lipid chain order parameters and counteracts the lipid condensation effect of cholesterol to some extent. Therefore, as the amount of cholesterol in the membrane increases, the membrane effect of [C_12_MIM]^+^Br^–^ decreases. The effect of [C_12_MIM]^+^Br^–^ on the membrane properties is more pronounced for POPC compared to that of POPG membranes. This suggests a dependence of these effects on the electrostatic interactions, indicating that the influence of [C_12_MIM]^+^Br^–^ varies based on the lipid composition. The findings suggest that the presence of cholesterol can modulate the effect of [C_12_MIM]^+^Br^–^ on membrane properties, with variations observed between POPC and POPG membranes, highlighting the importance of lipid composition. In short, this study provides insights into the intricate interplay between cholesterol, the lipid membrane, and the ionic liquid [C_12_MIM]^+^Br^–^.

## Introduction

Ionic liquids (ILs) are salts with a size disparity between cations and anions, causing imperfections in their crystalline packing. This results in the lowering of their melting point (<100 °C).^1^ The choice of ions provides synthetic control over the physiochemical^2^ and biological properties^3^ of ionic liquid by varying their structure. The tunable properties of ionic liquids make them the best candidates to be explored in wide applications in different research fields.^3,4^ Nonetheless, the extensively studied toxic properties of ionic liquids raise concerns about their usage.^5^ Among all of the ionic liquids, imidazolium-based ionic liquids are the most studied ionic liquids for various applications.^6^ Moreover, due to the imidazole moiety, these ionic liquids are biologically active.^5^ The plasma membrane of the cell is considered to be the first target of amphiphilic ionic liquids, which might cause an alteration in the cell metabolism after penetrating the cell leading to cell death. Therefore, their interaction with the lipid membranes is well studied to understand the mechanism of toxicity of ionic liquids.^5,7−16^ The observed interaction between ionic liquids and phospholipid membranes is closely correlated with cellular toxicity.^7−10,13^ Even since due to the complexity of cellular systems, it is challenging to pinpoint a single cause of cellular toxicity from ionic liquids. Therefore, simple phospholipid membranes are employed as close mimics of cellular membranes. There are only very few reports in the literature, where the interaction of ionic liquids with more complex phospholipid membrane systems has been studied.^11,15,17−21^

In this work, we are particularly interested in studying the interaction of ionic liquids with cholesterol-containing phospholipid membranes as cholesterol is known to play an important role in the functioning of eukaryotic cells and is found in the plasma membrane to an extent of 50 mol % total lipid content.^22^ It acts as a precursor molecule for the synthesis of steroidal hormones, bile salts, and vitamins.^23^ Interaction of cholesterol with various lipid components enhances the mechanical strength of membranes,^24,25^ regulates their fluidity,^26^ makes the membranes less permeable to water or small molecules,^27−29^ and also induces changes in the phase behavior of the membrane.^29,30^ Within a lipid bilayer, cholesterol induces the condensing effect^31−33^ by reducing the surface area per lipid molecule and increasing the order of lipid acyl chains. All of these (membrane) functions of cholesterol exhibit that it is important to study the membrane interaction of ILs with cholesterol-containing membranes since the cell toxicity of ILs will be influenced; on the other hand, ILs represent a potential drug delivery system and may have other biological applications.

In the literature, there are few reports in which the interaction of an ionic liquid was studied with cholesterol-containing model lipid membranes. Wiedmer et al. have studied the interaction of trioctylmethylphosphonium acetate ionic liquid with liposomes constituting PC with and without cholesterol using differential scanning calorimetry and nanoplasmonic sensing techniques.^17^ Similar studies were also conducted by the same group using the same ionic liquid on LUVs and MLVs made of eggPC/eggPG with and without cholesterol using small-angle X-ray scattering.^15^ They have found that the loss of phospholipid content by ionic liquids is slower in cholesterol-containing LUVs as compared to pure vesicles.

However, a systematic study of the impact of cholesterol in dictating the ionic liquid-induced membrane permeability and membrane fusion is still missing. Here, we studied the impact of cholesterol on the interaction of the [C_12_MIM]^+^Br^–^ ionic liquid with LUVs made of zwitterionic 1-palmitoyl-2-oleoyl-*sn*-glycero-3-phosphocholine (POPC) and anionic (1-palmitoyl-2-oleoyl-*sn-*glycero-3-phospho-(1′-rac-glycerol) (sodium salt)) (POPG) phospholipids using different molar ratios of cholesterol. Calcein-based dye leakage assays were performed to study the kinetics of membrane permeabilization. ζ-potential measurements were employed to determine the loading capacity/binding affinity of ionic liquids toward LUVs and dynamic light scattering (DLS) was used to study the size distribution of LUVs in the absence and presence of ionic liquids. ^2^H and ^31^P solid-state NMR studies were also performed to determine the structural impact of [C_12_MIM]^+^Br^–^ on cholesterol-containing bilayers. Further, a pressure–area isotherm study was also performed to look into the interaction of [C_12_MIM]^+^Br^–^ with cholesterol-containing POPC and POPG monolayers. The effect of higher concentrations of ionic liquid on cholesterol-containing POPC and POPG LUVs was also evaluated using DLS, Förster resonance energy transfer (FRET) pair-based probe dilution assay, and ζ-potential measurements. Finally, the discussion concluded on the role of cholesterol in governing the interaction between ionic liquid and lipid membranes, along with its impact on membrane permeability.

## Experimental Methods

### Materials

The powdered form of the phospholipids 1-palmitoyl-2-oleoyl-*sn*-glycero-3-phosphocholine (POPC) (>99%), 1-palmitoyl-2-oleoyl-*sn*-glycero-3-phospho-(1′-rac-glycerol) (sodium salt) (POPG) (>99%), as well as their *sn*-1 chain perdeuterated forms POPC-*d*_31_ and POPG-*d*_31_, cholesterol (Ovine wool, >98%), l-α-phosphatidylethanolamine-*N*-(lissamine rhodamine B sulfonyl) (ammonium salt) (Rho-PE) (>99%), and 1,2-dioleoyl-*sn*-glycero-3-phosphoethanolamine-*N*-(7-nitro-2–1,3-benzoxadiazol-4-yl) (ammonium salt) (NBD-PE) (>99%), was purchased from Avanti Polar Lipids, Inc. (Alabaster, AL). Anhydrous sodium phosphate monobasic (AR grade) (99%), calcein extrapure (AR grade), and Triton X-100 (molecular biology grade) were purchased from Sisco Research Laboratories Pvt., Ltd. (Maharashtra, India). Sephadex G-50 was purchased from Sigma-Aldrich, India. 1-Bromododecane (98%), 4-amino-3-hydroxy-1-naphthalene sulfonic acid (99%), and sodium dithionite (DTN) (>85%) were purchased from Alfa Aesar, India. Tris hydrochloride 99% (molecular biology grade), sodium chloride 99.5% (AR grade), and sulfuric acid 98% (AR grade) were purchased from Loba Chemie, Mumbai, India. Diethyl ether (AR grade) and sodium hydroxide pellets (AR grade) were purchased from SD Fine-Chem Limited, (Mumbai, India). Ammonium heptamolybdate tetrahydrate and sodium sulfite anhydrous (>98%) were purchased from Merck, India. Sodium metabisulfite (98.5%) was purchased from Fisher Scientific, India. 1-Methyl imidazole (>99%) was purchased from Spectrochem, India. Perchloric acid 70% (AR grade) was purchased from Qualikems Fine Chem Pvt. Ltd., India. Ionic liquid [C_12_MIM]^+^Br^–^ was synthesized as per the previous report^13^ and its characterization and synthesis details were also shown.

### Methods

#### Preparation of LUVs

For the dye leakage, dynamic light scattering, ζ-potential, and lipid mixing measurements, POPC and POPG phospholipid-based LUVs containing variable amounts of cholesterol were prepared. For the preparation of LUVs, a 5 mM solution of POPC and POPG along with 10, 20, 30, and 40 mol % cholesterol was dissolved in chloroform. A thin film was formed on the walls of a glass vial by removal of chloroform under a gentle stream of nitrogen. To further remove any residual chloroform, the sample was dried overnight under a vacuum. The rest of the protocol was similar to our previous report.^13^

#### Membrane Permeability Assay

Membrane permeability assay was performed on the POPC and POPG LUVs composing variable amounts of cholesterol (0, 10, 20, 30, 40 mol %) encapsulated with self-quenched calcein dye at a 70 mM concentration. A stock solution of [C_12_MIM]^+^Br^–^ (100 mM) was prepared in 7.7 mM Tris HCl buffer containing 100 mM NaCl (pH 7.4). An appropriate amount of dye-filled cholesterol-containing POPC and POPG LUVs was added to the buffer containing [C_12_MIM]^+^Br^–^ to achieve a final phospholipid concentration of 0.275 ± 0.015 mM in each case. The remaining procedure for dye leakage measurement is similar to that described earlier.^13^ The solutions were gently mixed and transferred to a quartz cuvette to perform fluorescence measurements (dead time = 30 s) using a PerkinElmer LS-55 Luminescence spectrometer. Calcein emission was measured at 520 nm with the excitation wavelength set at 485 nm using an excitation and emission slit width of 10 nm each. The percentage of dye leakage was calculated by using the following equation
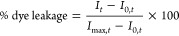
where *I*_0,*t*_ and *I_t_* are the observed fluorescence intensities in the absence and presence of ionic liquids and *I*_max,*t*_ is the fluorescence intensity obtained after the addition of 1% Triton X-100. A separate set of controls *I*_max,*t*_ and *I*_0,*t*_ were recorded for each LUV preparation. The normalized data was smoothened by three-point averaging and plotted against time. All of the measurements were repeated thrice, and the reproducibility of results was obtained with a standard deviation of less than 3%.

#### Dynamic Light Scattering (DLS) and ζ-Potential Measurements

A stock solution of 100 mM [C_12_MIM]^+^Br^–^ ionic liquid was prepared in 7.7 mM Tris HCl buffer containing 100 mM NaCl (pH 7.4). Size distribution measurements of cholesterol-containing POPC and POPG LUVs were performed in the absence and presence of variable concentrations (1–10 mM) of [C_12_MIM]^+^Br^–^ ionic liquid at 25 °C using a Malvern instrument (Zeta-sizer, Nano Series, nano-ZS, Malvern, U.K.). Samples were thermally equilibrated for 10 min before each measurement. The concentration of phospholipids in LUVs was fixed at 0.275 ± 0.015 mM in each measurement. ζ-potential measurements of cholesterol-containing POPC and POPG LUVs were measured in the absence and presence of variable concentrations (0.05, 0.2, 0.5, 0.75, and 1 mM) of [C_12_MIM]^+^Br^–^ at 25 °C. The ζ-potential measurements of cholesterol-containing POPC and POPG LUVs were also performed in the presence of a higher concentration of [C_12_MIM]^+^Br^–^ (1–10 mM). All of the measurements were performed in the triplicate and the results of ζ-potential are reported along standard deviation. In the DLS measurements, the standard deviation was less than 5%.

#### Lipid Mixing Assay

To measure lipid mixing during membrane fusion, a probe dilution assay based on the mixing of LUVs containing FRET pairs was performed.^12^ The POPC and POPG LUVs were composed of variable amounts (0, 10, 20, 30, 40 mol %) of cholesterol, which also contains FRET pair probes (NBD-PE (donor) and Rho-PE (acceptor)) at a concentration of 1.5 mol % each. The rest of the protocol is similar to that described earlier.^12^ Fluorescence dequenching of NBD-PE due to dilution of FRET probes into probe-free LUVs was monitored after 10 min of the addition of different concentrations of ionic liquids. The percentage of lipid mixing was calculated by using the following equation:
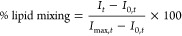
where *I_t_* is the fluorescence emission intensity of NBD-PE at time *t* in the presence of ionic liquids, and *I*_0,*t*_ is the fluorescence intensity in the absence of ionic liquids. *I*_max,*t*_ is the maximum fluorescence intensity obtained after the addition of 1% (v/v) Triton X-100. A correction factor of 1.5 was applied to observed fluorescence in the last case as Triton X-100 is known to affect NBD-PE fluorescence.^12^ All of the measurements were performed thrice, and all of the results were reproducible with a standard deviation of less than 5%.

#### Sample Preparation for Solid-State NMR Studies

The lipids and [C_12_MIM]^+^Br^–^ were mixed in the desired molar ratios in chloroform. The solvents were evaporated, and after redissolving the obtained lipid film in cyclohexane, the samples were lyophilized overnight at high vacuum. The obtained fluffy powders were hydrated with 50 wt % Tris·HCl buffer (7.7 mM Tris·HCl and 100 mM NaCl) and homogenized by ten freeze–thaw cycles. Finally, the samples were packed in 4 mm HR MAS rotors with spherical Kel-F inserts for NMR measurements to seal the sample properly.

#### ^2^H NMR and ^31^P NMR Spectroscopy

The static ^2^H NMR studies were acquired on a Bruker DRX300 NMR spectrometer (Bruker Biospin GmbH, Rheinstetten, Germany) with a phase-cycled quadrupolar echo sequence. The two π/2 pulses with a length of ∼3.2 μs were separated by a 50 μs delay, and the relaxation delays were 1 s. About 20,000 scans were performed. The obtained spectra were dePaked,^34^ and the smoothed order parameter profiles were calculated according to Lafleur et al.^35^

The ^31^P NMR experiments were conducted on a Bruker Avance III 600 MHz spectrometer using a Hahn echo sequence with a relaxation delay of 2.5 s. The 90° pulse was 10 μs. Low-power ^1^H decoupling was applied. The number of scans was 8k.

All of the experiments were performed at 298 K.

#### Pressure–Area (PA) Isotherm Measurements

##### Thermodynamic Techniques

To investigate the thermodynamic parameters of the interaction between the ionic liquid (IL) and lipids, a Langmuir–Blodgett (LB) trough of size 36.4 × 7.5 × 0.4 cm^3^ (KSV NIMA, Biolin Scientific) with two symmetric Delrin barriers and a platinum Wilhelmy balance was used to record the isotherms of the monolayers. The monolayers of lipids, cholesterol, ionic liquid, and mixed system (lipid/IL, lipid/cholesterol, and lipid/cholesterol/IL) were formed at the air–water interface. All samples of lipid, cholesterol, and the ionic liquid (IL) were individually dissolved in chloroform to prepare a stock solution of 0.5 mg/mL. Subsequently, the solutions of ILs and lipids were combined to achieve a specific mole percentage (mol %). The amount of IL in the lipid/IL mixture was quantified as
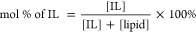
1Here, [IL] and [Lipid] are the molar concentrations of IL and lipid, respectively.

##### Surface Area–Pressure Isotherm

To generate an isotherm at a specific temperature, the surface pressure was monitored as a function of the mean molecular area. This is a highly sensitive surface characterization technique, hence requiring rigorous cleaning of the trough with ethanol and deionized (DI) water. A 40 μL lipid or lipid/IL solution was spread on the water surface of the trough using a glass Hamilton microsyringe, followed by a 20 min waiting time to allow the complete evaporation of the solvent. The monolayer was compressed at a consistent rate of 4 mm/min until it reached the collapse pressure. All of the isotherms were recorded at 25 °C by circulating water using a water bath (Equibath, India).

##### Membrane In-Plane Elasticity

During the quasistatic process of compression of barriers, molecules are considered to be in equilibrium and steady state. The in-plane elasticity of the monolayer is evaluated using the isotherm itself, therefore known as static elasticity. It depends upon the rate of change in surface pressure with the change in surface area at a constant temperature and is calculated using the following relation^36,37^
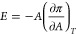
2Here, *A* denotes the mean molecular area, whereas π is the lateral surface pressure at a given temperature (*T*). This static elasticity is also termed as the compressional modulus.

##### Gibbs Free Energy

The excess Gibbs free energy (Δ*G*_exc_) is calculated by using the same isotherm and does not require any additional measurements. However, the only requirement is that all of the components must exhibit an isotherm. The following equation is used to evaluate the Gibbs free energy from isotherm^7,36,38^
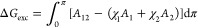
3Here, χ_1_ and χ_2_ are the mole fractions of two components used in a binary system. *A*_12_ is the experimentally estimated mean molecular area (MMA) or the area per molecule in the monolayer of a binary system. *A*_1_ and *A*_2_ are the MMA of both components calculated from their individual isotherms. According to the region or thermodynamic phase of interest, the limit of integration over a pressure range can be chosen.

## Results and Discussion

### Membrane Permeability Assay

**Figure 1:**
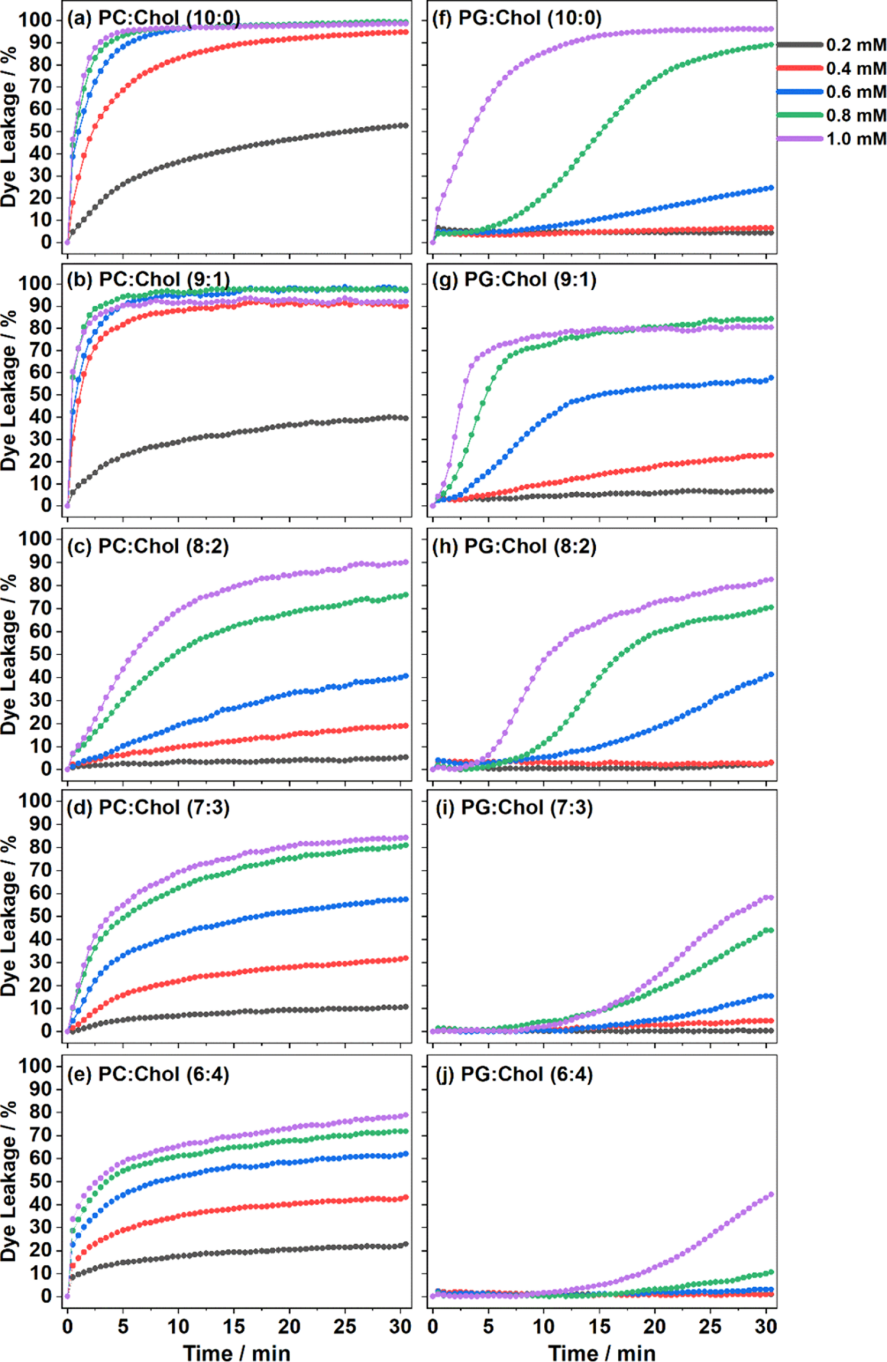
Time-based dye leakage in the presence of 0.2, 0.4, 0.6, 0.8, and 1 mM [C_12_MIM]^+^Br^–^ from (a) PC/Chol (10:0), (b) PC/Chol (9:1), (c) PC/Chol (8:2), (d) PC/Chol (7:3), (e) PC/Chol (6:4), (f) PG/Chol (10:0), (g) PG/Chol (9:1), (h) PG/Chol (8:2), (i) PG/Chol (7:3), and (j) PG/Chol (6:4) LUVs measured at 25 °C. These measurements were performed 3 times, and plots are the average of obtained data from three experiments. The experimental error for all of these measurements is less than 3%. The dye leakage data for PC/Chol (10:0) and PG (10:0) in the presence of [C_12_MIM]^+^Br^–^ are adapted from our previous publication.^11^

Owing to the surface-active behavior of imidazolium-based ionic liquids, they easily intercalate into the lipid membrane and show a detergent-like effect. This causes alteration in the membrane integrity and stability, which might lead to membrane permeabilization.^7−13,39^ Membrane permeability can be easily determined by measuring the amount of dye leaking out from cholesterol-containing POPC and POPG LUVs as a function of [C_12_MIM]^+^Br^–^ concentration and time. Figure 1a–j shows time-based leakage of calcein dye from the PC/Chol (10:0, 9:1, 8:2, 7:3, 6:4) and PG/Chol (10:0, 9:1, 8:2, 7:3, 6:4) LUVs as a function of variable [C_12_MIM]^+^Br^–^ concentration. In our previous publication, we have shown that POPC LUVs are more leakage-prone than POPG LUVs.^14^ A similar trend is observed in PC/Chol and PG/Chol LUVs. The impact of cholesterol on LUV permeability is more pronounced in the case of POPG LUVs than that of POPC LUVs.

The resistance of POPC and POPG membranes against the ionic liquid increases with an increase in the amount of cholesterol. The POPC or POPG/Chol (9:1) is the only exception to this trend, where membrane permeability increases on some of the studied concentrations of ionic liquid (Figure 1). Similar kinds of results were also observed in pure PC and PC/Chol LUVs in the presence of N-9 (nonionic), C31G (an amphoteric mixture of two surface-active molecules), C14 alkylamine oxide and C16 alkyl betaine (zwitterionic), BZK (cationic), and SDS (anionic) surfactants studied by Apel-Paz et al.^40^ The time-based leakage kinetics of PC/Chol and PG/Chol LUVs after the addition of 0.6 mM [C_12_MIM]^+^Br^–^ (Figure S1) was fitted with a sigmoidal equation (% dye leakage = *a*_0_/(1 + e^–(*t*–*t*_c_)×*k*^)), which provides the rate constant *k*, maximum dye leakage (*a*_0_), and time (*t*_c_), at which dye leakage reduced to *a*_0_/2, as shown in Table 1. In PC/Chol LUVs, the rate of leakage decreases with an increase in cholesterol content, but in POPG LUVs containing 10 mol % cholesterol, the rate of leakage is faster than in pure POPG LUVs. With a further increase in cholesterol content to 20 mol % and above the rate of leakage decreases (Table 1 and Figure 1). This reduction in the extent of dye leakage on the addition of cholesterol in POPC and POPG LUVs might be due to an increase in rigidity of the lipid bilayer,^41^ which makes the membrane less permeable to the ionic liquid. Cholesterol is also known to make the membrane more rigid by increasing the order of the lipid acyl chain as also seen in the ^2^H NMR measurements.^42^ This effect may also contribute to the lower permeability of cholesterol-containing POPC and POPG LUVs.

**Table 1:** Parameters Defining the Leakage Kinetics, and Change in ζ-Potential of PC/Chol and PG/Chol LUVs in the Presence of 0.6 mM [C_12_MIM]^+^Br^–^ Ionic Liquid^a^^13^

system	*k* (min^–1^)	*a*_0_ (%)	*t*_c_ (min)	Δζ (mV)
PC/Chol (10:0)	0.52 ± 0.02	99.1 ± 1.48	0.87 ± 0.07	35.7 ± 3.0
PC/Chol (9:1)	0.55 ± 0.03	98.6 ± 1.23	0.41 ± 0.12	14.5 ± 1.0
PC/Chol (8:2)	0.17 ± 0.01	40.7 ± 0.55	11.47 ± 0.12	15.1 ± 0.2
PC/Chol (7:3)	0.18 ± 0.01	57.3 ± 1.00	4.94 ± 0.26	19.5 ± 3.7
PC/Chol (6:4)	0.17 ± 0.01	62.0 ± 1.24	0.98 ± 0.24.	18.2 ± 2.4
PG/Chol (10:0)	0.15 ± 0.01	24.60 ± 0.34	16.21 ± 0.20	37.0 ± 2.4
PG/Chol (9:1)	0.30 ± 0.01	57.75 ± 0.78	8.20 ± 0.12	40.7 ± 0.2
PG/Chol (8:2)	0.20 ± 0.02	41.42 ± 0.86	20.60 ± 0.16	30.5 ± 2.2
PG/Chol (7:3)	0.28 ± 0.01	15.32 ± 0.45	22.73 ± 0.160	49.8 ± 4.5
PG/Chol (6:4)	N.D.	3.05 ± 0.03	N.D.	50.8 ± 1.7

a N.D. refers to the value that was too high or too low to yield meaningful fitting results. The Δζ values are reported for 1 mM [C_12_MIM]^+^Br^–^. The change Δζ for PC/Chol (10:0) and PG/Chol (10:0) LUVs are adapted from our previous publication.^13^

### Lipid Headgroup and Lipid Chain Dynamics

For further understanding of the leakage behavior of cholesterol-containing membranes, the impact of the insertion of the amphiphilic cation on the lipid bilayer, and how cholesterol modifies these interactions, solid-state NMR measurements were performed. To check the phase state of the lipid bilayer and to obtain information about the dynamics of the lipid headgroup, ^31^P NMR spectra of the samples with different cholesterol amounts in the pre- and absence of [C_12_MIM]^+^Br^–^ were acquired. All spectra (not shown) exhibit the typical line shape of a liquid-crystalline lamellar phase. The chemical shift anisotropy (CSA, Δσ) given by the width of the spectra depends on the orientation and dynamics of the phospholipid head groups.^43,44^ Therefore, CSA values help to estimate the impact of the insertion of amphiphilic cations into the lipid bilayer as reported earlier by Seelig et al.,^43,44^ Kumar et al.,^11,13,14^ and Kaur et al.^7−9^ Insertion of a cation can impact the wobbling motion of the phospholipid headgroup or can lead to changes in the molecular orientation of the ^–^P–^+^N dipole, which is termed as the molecular voltmeter effect.^43,44^ For all investigated lipid membranes, the CSA values are significantly increased in the presence of [C_12_MIM]^+^Br^–^ as was observed before for POPC and POPG membranes without cholesterol.^14^ There is no dependence of this effect on the amount of cholesterol in the phospholipid membrane. This suggests that the effects of headgroup orientation (voltmeter effect) are not influenced by the absence or presence of cholesterol.

**Figure 2:**
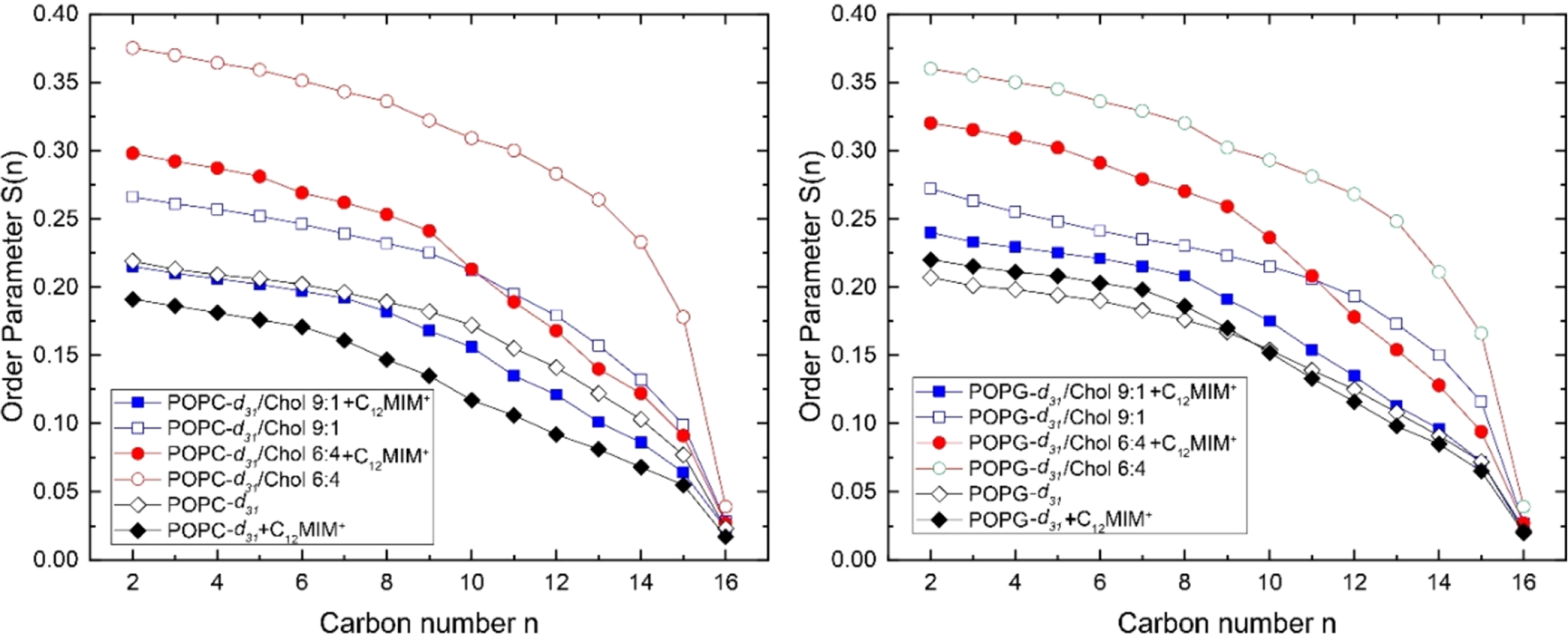
^2^H NMR chain order parameter of POPC-*d*_31_ (left) and POPG-*d*_31_ (right) with a different amount of cholesterol in the lipid bilayers in the absence (open symbols) and the presence (closed symbols) of [C_12_MIM]^+^. The chain order parameters of POPC-*d*_31_ and POPG-*d*_31_ in the pre- and absence of [C_12_MIM]^+^Br^–^ are adapted from our previous publication.^14^

^2^H NMR was used to investigate the influence of [C_12_MIM]^+^Br^–^ on the molecular order and dynamics of lipid chains in the hydrocarbon core of the lipid membrane. Figure 2 shows the smoothed chain order parameter plots for POPC or POPG with a perdeuterated *sn*-1 palmitoyl chain in the absence or presence of [C_12_MIM]^+^Br^–^ for different amounts of cholesterol in the membrane. As expected, the order parameters are increased due to the addition of cholesterol and a liquid-ordered phase is formed.

In the absence of cholesterol, the addition of [C_12_MIM]^+^Br^–^ in POPC-*d*_31_ membranes leads to decreased chain order parameters.^14^ This trend continues and is enhanced for POPC-*d*_31_/cholesterol membranes, where the IL seems to counteract the lipid condensation effect of cholesterol to some extent but not completely. These largely decreased order parameters are also reflected in the average chain order parameter and the calculated lipid chain length^45^ (*L*_c_*) given in Table 2.

**Table 2:** Lipid Chain Extent *L*_c_*,^46^ Average Chain Order Parameter and ^31^P NMR Chemical Shift Anisotropy Δσ for Cholesterol-Containing POPC and POPG Bilayers in the Absence and Presence of [C_12_MIM]^+^^a^^14^

	*L*_c_* (Å)		⟨*S*⟩		Δσ (ppm)	
membrane	+ [C_12_MIM]^+^	w/o	+ [C_12_MIM]^+^	w/o	+ [C_12_MIM]^+^	w/o
POPC	10.5	11.8	0.125	0.161	63.9	45.9
POPC/Chol 9:1	11.4	12.8	0.151	0.198	64.0	48.0
POPC/Chol 6:4	13.0	14.9	0.209	0.295	63.0	43.0
POPG	12.2	11.4	0.152	0.148	42.9	35.0
POPG/Chol 9:1	11.9	13.0	0.169	0.203	43.0	38.0
POPG/Chol 6:4	13.3	14.7	0.224	0.280	44.0	37.0

a The values for membranes without cholesterol were taken from our previous publication.^14^

While for pure POPG-*d*_31_ membranes, the chain order parameters are overall only slightly influenced by the presence of [C_12_MIM]^+^Br^–^, and they are also decreased in POPG-*d*_31_/cholesterol membranes, albeit to a much lesser extent than for the respective POPC-*d*_31_/cholesterol membranes. Therefore, for these membranes, more of the lipid condensation effect of cholesterol is “conserved”.

These observations are a hint to explain lower leakage in the presence of higher amounts of cholesterol, especially for POPG membranes.

### Pressure–Area Isotherm Measurements

**Figure 3:**
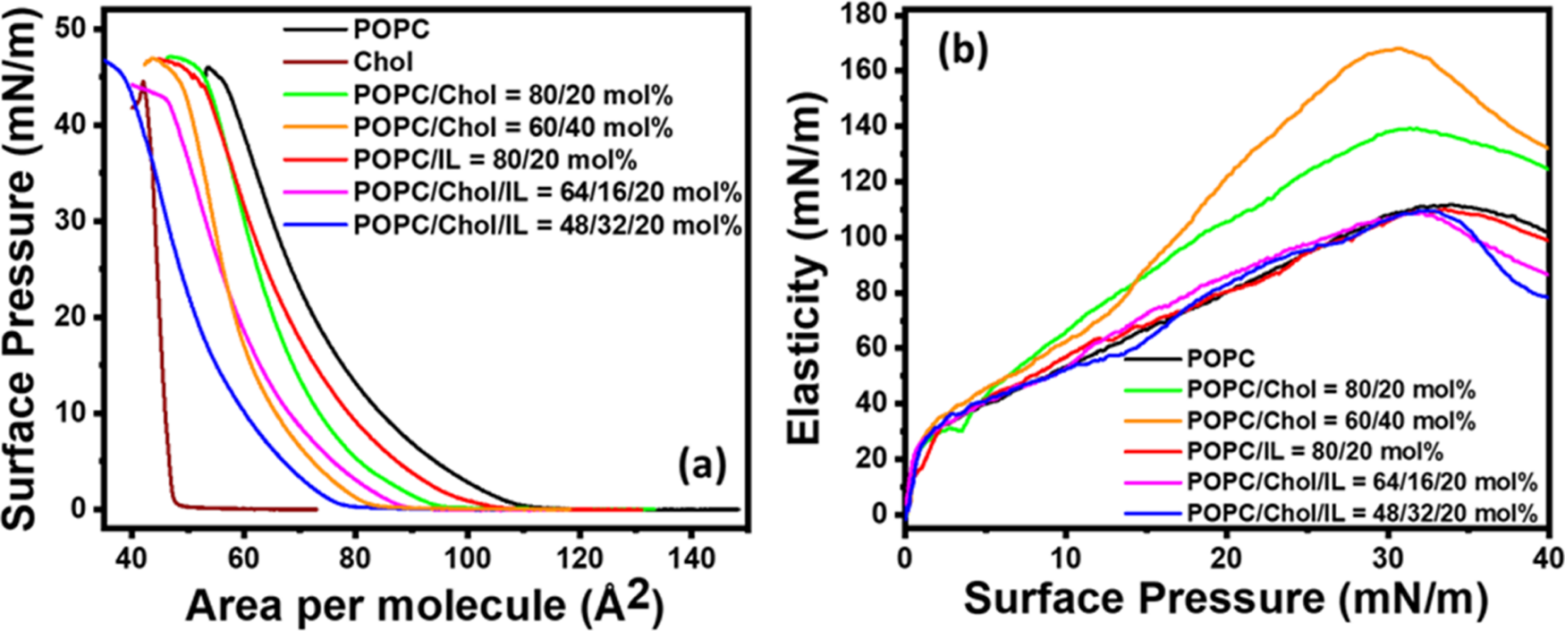
(a) Surface pressure–area isotherm of pure POPC lipid and mixed system consists of POPC, cholesterol, and ionic liquid monolayers formed at the air–water interface and measured at 25 °C. (b) Exhibits the corresponding in-plane elasticity of the respective monolayer.

**Figure 4:**
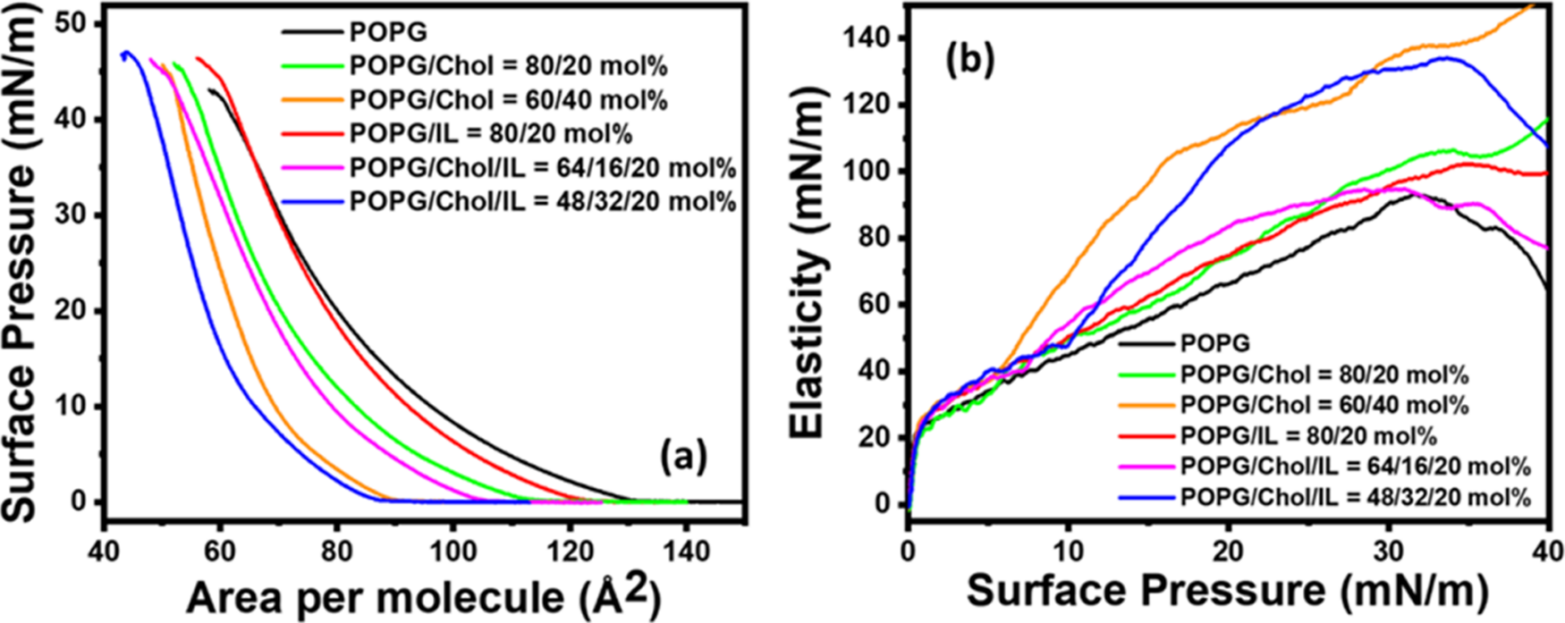
(a) Surface pressure–area isotherm of pure POPG lipid and mixed system consists of POPG, cholesterol, and ionic liquid monolayers formed at the air–water interface and measured at 25 °C. (b) Exhibits the corresponding in-plane elasticity of the respective monolayer.

One of the leaflets of a cell membrane can be mimicked by the self-assembly of phospholipids as a monolayer on the air–water interface. The measurement of changes in surface pressure is a well-known method to estimate the thermodynamic parameters for the interaction of ILs with lipids.^47−50^Figures 3a and 4a show pressure–area (PA) isotherms of POPC and POPG with varying molar ratios of cholesterol and ionic liquids. The effect of the addition of [C_12_MIM]^+^Br^–^ is also observed on the PA isotherms of the lipid monolayers constituting different compositions of lipids and cholesterol molecules. Both lipids exhibit a smooth transition from the liquid-extended (LE) to liquid-condensed (LC) phase, and there is no signature of the coexisting plateau region. Saturated lipids, such as DPPC and DPPG, exhibit a first-order phase transition from the LE to LC phase, exhibiting coexisting of the LE-LC phase as a plateau region in the PA isotherm.^36^ This is not the case in the study presented here with POPC and POPG phospholipids. Static elasticity and excess Gibbs free energy have been calculated from the same isotherms using eqs 2 and 3, respectively. Lift-off area and Δ*G*_exc_ values calculated over a pressure range of 0 to 30 mN/m are shown in Table 3. Recently, the surface activity and interaction of an IL with POPC and POPG lipids have been reported in the literature.^7^ POPG is a charged lipid; hence, molecules experience an electrostatic repulsion and occupy a higher area than the zwitterionic lipid POPC as shown in Figures 3 and 4. The lift-off area values are ∼132 and ∼111 Å^2^ for POPG and POPC, respectively. These values are close to the reported values in the literature.^36,51^ This lift-off area provides information about the interaction between molecules when they are in the LE phase and just start interacting. There is a consistent reduction of area for both lipids after the addition of 20 and 40 mol % cholesterol.

**Table 3:** Lift-Off Area and Excess Gibbs Free Energy Calculated for a Mixed System Composed of Lipids, Cholesterol, and Ionic Liquid

system	lift-off area (Å^2^)	Δ*G*_exc_ (J mol^–1^)
ionic liquid (IL)	15	
POPC	111	
POPG	132	
cholesterol	48	
POPC/Chol = 80/20 mol %	97	–71.31
POPC/Chol = 60/40 mol %	84	–86.38
POPC/IL = 80/20 mol %	107	254.63
POPC/Chol/IL = 64/16/20 mol %	90	123.56
POPC/Chol/IL = 48/32/20 mol %	79	53.51
POPG/Chol = 80/20 mol %	113	–84.94
POPG/Chol = 60/40 mol %	90	–156.44
POPG/IL = 80/20 mol %	124	381.94
POPG/Chol/IL = 64/16/20 mol %	106	244.56
POPG/Chol/IL = 48/32/20 mol %	87	98.35

This is the condensing effect of cholesterol, in which lipid molecules are assembled in a compact arrangement enhancing the surface elasticity, also known as compressional modulus.^52^ Interestingly, Δ*G*_exc_ is negative for both lipids in the presence of cholesterol but attains a more negative value for POPG.^53^ The addition of 20 mol % IL in the membrane slightly enhances the elasticity of the POPG membrane with a positive Δ*G*_exc_ (381.94 J mol^–1^) (Table 3). However, there is no drastic change in the elasticity of the POPC membrane, and Δ*G*_exc_ attains a positive value of 254.63 J mol^–1^. ILs are reported to disorder the lipid membrane as explained in multiple recent studies.^5,7,8,38^ Both electrostatic interactions and hydrophobic interactions play a role in the interaction between the IL and lipid films, where adsorption is primarily influenced by electrostatics, while insertion depends on the hydrophobic nature of the molecules.

As mentioned above, the presence of cholesterol in the membrane enhances the elasticity by compacting the lipid film, which may cause a reduction of the permeability of the membrane. It may then restrict the physical insertion of ILs into the lipid film, leading to a lesser destructive effect of the ionic liquid (Table 3). Interestingly, the addition of cholesterol in the presence of IL reduced the net Δ*G*_exc_ for both the lipids, and this decrease is systematic with the increase in cholesterol concentration. The amounts of reduction in Δ*G*_exc_ for the POPG/Chol/IL system are ∼35% (for 20 mol % cholesterol) and ∼74% (for 40 mol % cholesterol), which are relatively less than those for the POPC/Chol/IL system. The corresponding values for POPC/Chol/IL systems are ∼48% (for 20 mol % cholesterol) and ∼79% (for 40 mol % cholesterol) (Table 3). These results of the restricting effect of cholesterol in inserting the IL into lipid membranes are consistent with the membrane permeability results.

### Binding Affinity of [C_12_MIM]^+^Br^–^ toward Cholesterol-Containing POPC and POPG LUVs

**Figure 5:**
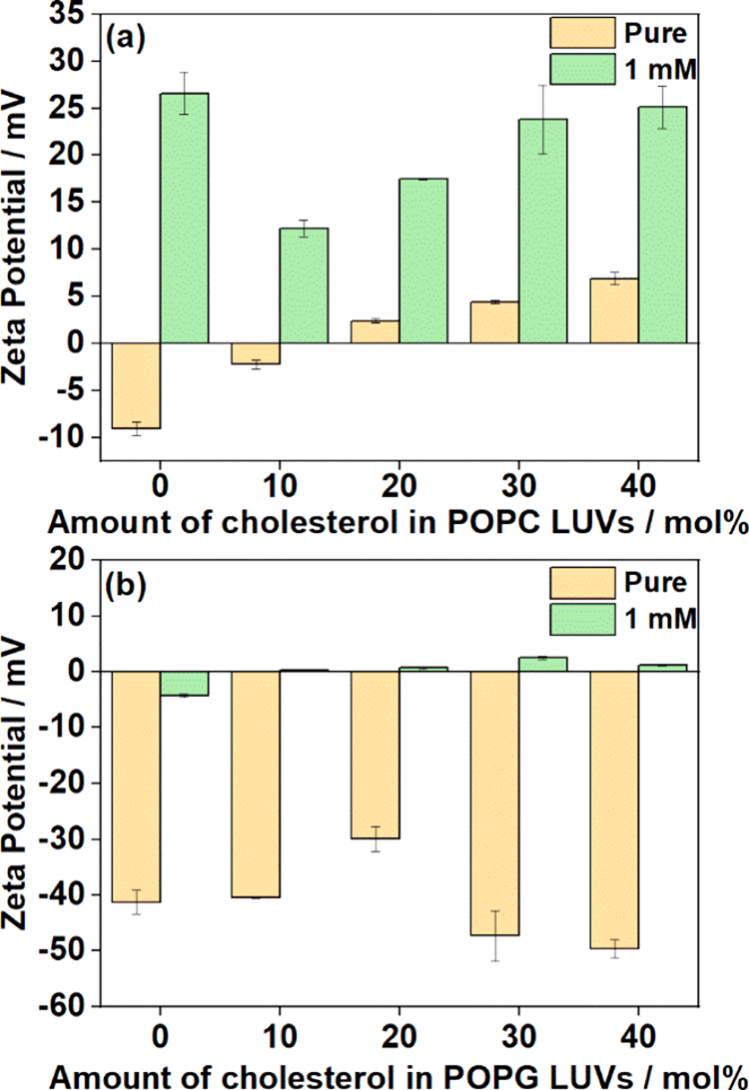
ζ-potential of (a) PC/Chol and (b) PG/Chol LUVs in the absence and presence of 1 mM [C_12_MIM]^+^Br^–^ at 25 °C. The ζ-potential values of POPC and POPG LUVs in the pre- and absence of 1 mM [C_12_MIM]^+^Br^–^ are adapted from our previous publication.^13^

Figure 5a,b shows the change in ζ-potential of cholesterol-containing POPC and POPG LUVs in the absence and presence of 1 mM [C_12_MIM]^+^Br^–^. The addition of a positively charged [C_12_MIM]^+^ cation into the LUVs increases the ζ-potential. The absolute change in ζ-potential values provides information about the loading capacity/binding affinity of ionic liquid toward LUVs. The overall change in ζ-potential values (Δζ) of PC/Chol LUVs in the presence of 1 mM [C_12_MIM]^+^Br^–^ decreases with an increase in cholesterol content (Table 1). This means that the loading capacity/binding affinity of ionic liquid toward PC/Chol LUVs decreases with an increase in cholesterol content. This is in agreement with the observations for the membrane permeability, which also decreases with increasing cholesterol content.

Seemingly, the loading capacity of PG/Chol (7:3 and 6:4) LUVs is more than that of other PG/Chol (10:0, 9:1, 8:2) LUVs. LUVs contain a lesser amount of cholesterol content. Instead, POPC LUVs offer lower affinity to [C_12_MIM]^+^ cations when the cholesterol content is higher. Moreover, given the fact that POPG LUVs containing high cholesterol content are less prone to leakage, this observation once again underlines the fact that binding is not a primary factor that dictates membrane permeability.^14^ The electrostatic interactions are also known to play a crucial role in dictating vesicle–vesicle interactions in the presence of ionic liquids.^12^ High-charged vesicles cause electrostatic repulsions among the vesicles, which decrease the probability of vesicle fusion. For membrane fusion, the vesicles should come close enough to make the initial point of contact but a higher electrostatic charge (positive or negative) prevents this.^12^ Based on the electrostatic interactions, POPC LUVs were found to be less fusion-prone than POPG LUVs in the presence of ionic liquids.^12^

### Impact of Higher Concentration of [C_12_MIM]^+^Br^–^ on the Size Distribution of Cholesterol-Containing POPC and POPG LUVs

**Figure 6:**
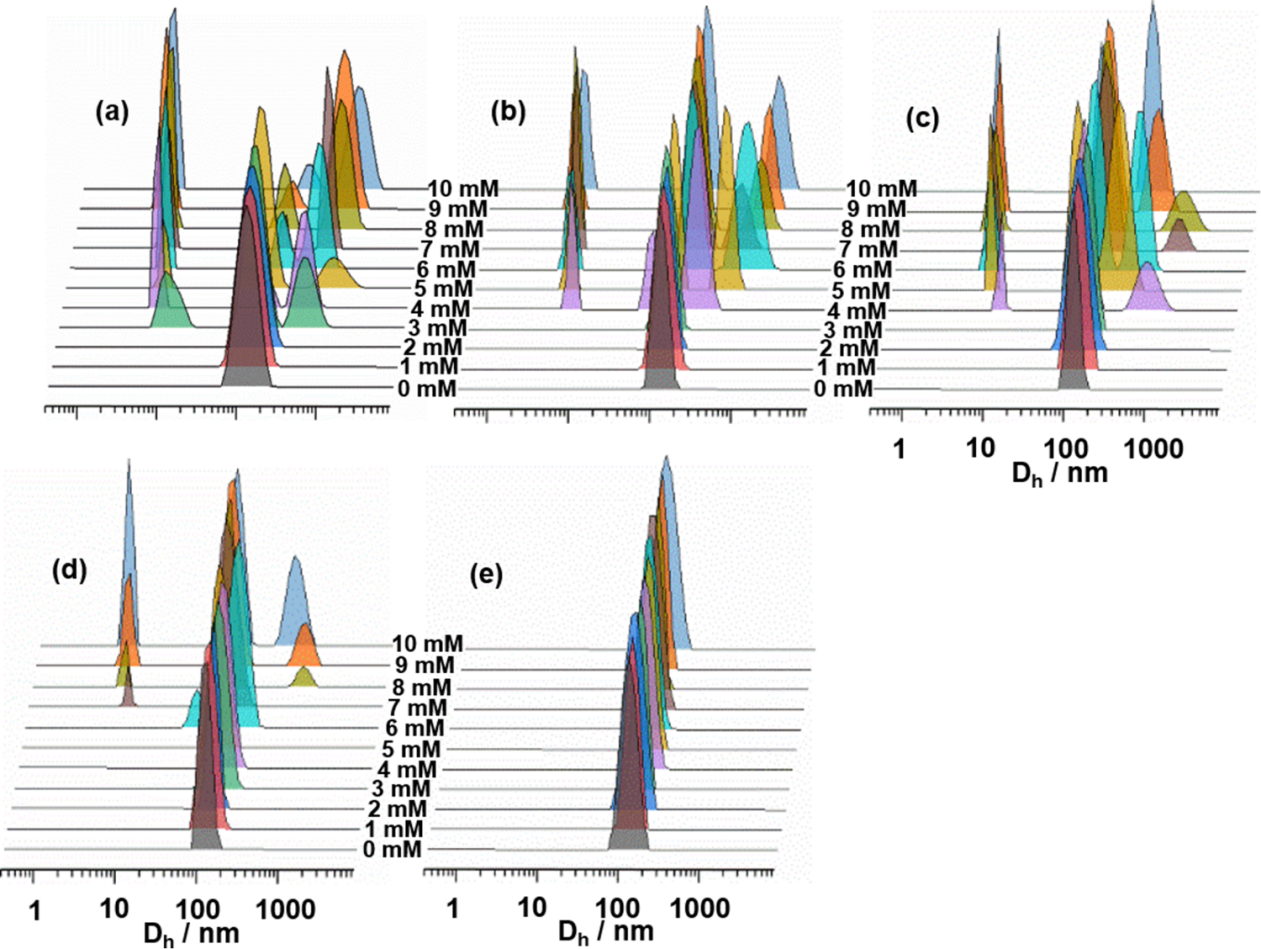
Hydrodynamic diameter (*D*_h_) of (a) PC/Chol (10:0), (b) PC/Chol (9:1), (c) PC/Chol (8:2), (d) PC/Chol (7:3), and (e) PC/Chol (6:4) LUVs at 25 °C after the addition of [C_12_MIM]^+^Br^–^ at the indicated concentrations. Total phospholipid concentration in the LUVs is 0.275 mM in all of the cases. The hydrodynamic diameter of PC/Chol (10:0) in the pre- and absence of [C_12_MIM]^+^Br^–^ is adapted from our previous publication.^12^

Next, we monitored the size distribution of cholesterol-containing LUVs as a function of a variable concentration of [C_12_MIM]^+^Br^–^ using DLS (Figures 6 and S2). The average hydrodynamic radius of the studied LUVs falls within the range of approximately 100–125 nm. An increase in size is observed upon the addition of ionic liquid but only after reaching a certain concentration, which varies depending on the lipid composition. For example, at 3 mM, new peaks corresponding to particles sized around 1110 nm are detected in pure POPC.^12^ Similar kinds of results were also observed in DOPC/SM vesicles in the presence of [C_12_MIM]^+^ cations, and due to the fusion of vesicles, the size of LUVs increased from 100 nm to 1.7 μm.^54^ Therefore, the larger peaks observed in PC/Chol and PG/Chol LUVs correspond to the fused or aggregated vesicles. In our previous report,^12^ we have shown that this increase in the size of LUVs was due to the fusion of LUVs and not due to aggregation. Additionally, increasing the amount of cholesterol also impedes vesicle fusion. The addition of an ionic liquid has a significantly low to negligible effect on the dynamic light scattering (DLS) profile of PC and PG LUVs containing the highest ratio of cholesterol studied here.

Besides peaks corresponding to large-sized particles, peaks at smaller size distributions in the range ∼5 to 50 nm were also observed in the DLS profiles of PC/Chol and PG/Chol LUVs, which correspond to micelles of [C_12_MIM]^+^Br^–^ or mixed [C_12_MIM]^+^/lipid micelles. In the presence of ionic liquids, the formation of mixed micelles of ionic liquid and lipids has been already reported previously in the literature.^12,16^ The formation of mixed micelles might be due to the interaction of [C_12_MIM]^+^Br^–^ micelles with liposomes, which results in population exchange among the micellar and lipidic phases. The results of DLS measurements confirm that the cholesterol-containing POPC and POPG LUVs undergo fusion in the presence of high ionic liquid concentrations.

### Lipid Mixing Assay

**Figure 7:**
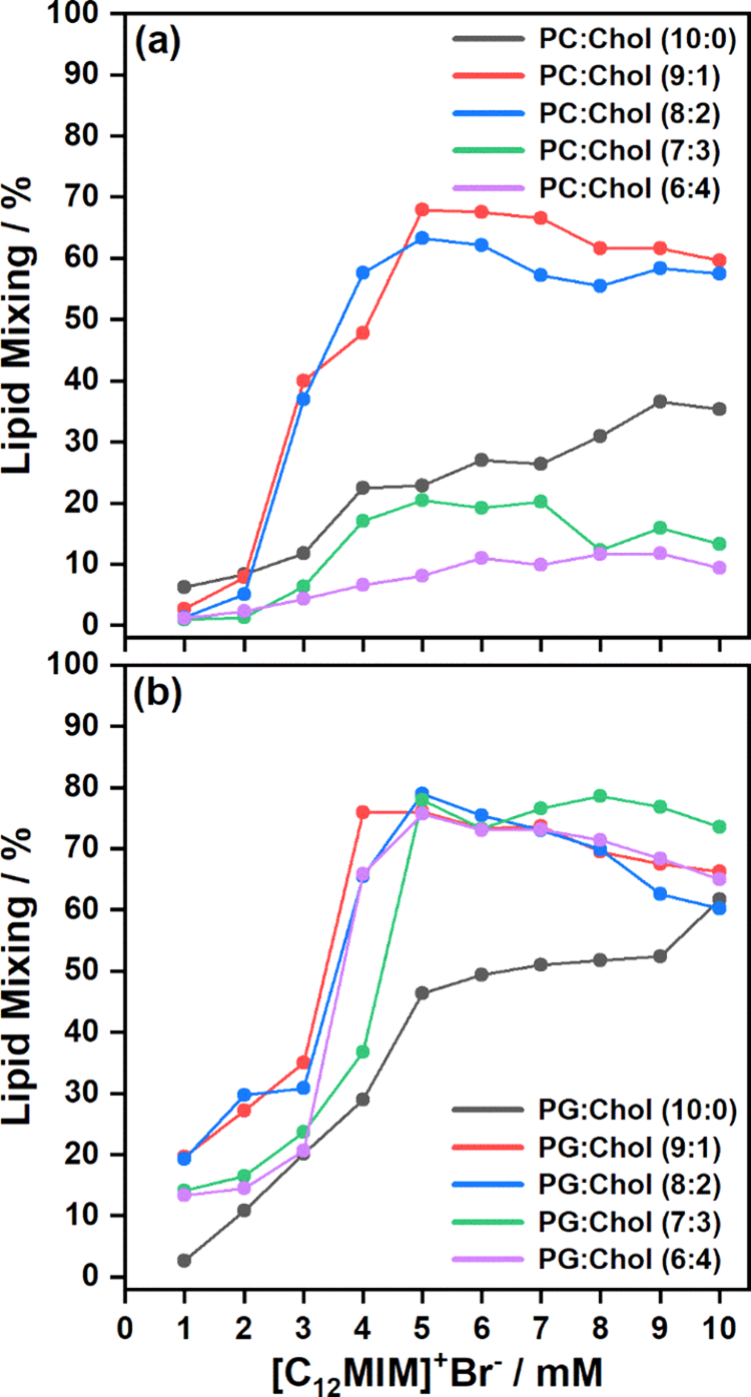
Extent of lipid mixing in (a) PC/Chol and (b) PG/Chol LUVs after 10 min of addition of a variable concentration of [C_12_MIM]^+^Br^–^ at 25 °C. The extent of lipid mixing for PC/Chol (10:0) and PG/Chol (10:0) LUVs in the presence of [C_12_MIM]^+^Br^–^ is adapted from our previous publication.^12^

To cross-check the possibility of the formation of fused vesicles as indicated by DLS measurement, we have performed a fluorescence-based “probe dilution” assay to measure the extent of lipid mixing among the merging LUVs as a function of increasing ionic liquid concentration.^55^ In this assay, PC/Chol and PG/Chol LUVs containing 1.5 mol % FRET pairs (NBD-PE and Rho-PE) were mixed with probe-free LUVs in the molar ratio of 1:4. Dilution of fluorescent probes in the membrane due to vesicle fusion leads to a decrease of the FRET efficiency, which was monitored as a function of [C_12_MIM]^+^Br^–^ concentration. The extent of lipid mixing in PC/Chol and PG/Chol LUVs in the presence of a variable concentration of ionic liquids after 10 min is shown in Figure 7a,b, respectively. The extent of lipid mixing confirms that the higher concentration of ionic liquid induces membrane fusion. When the amount of cholesterol is low (10 and 20 mol %) in POPC LUVs, the extent of lipid mixing is almost 3 times that of pure POPC LUVs. With a further increase in the concentration of cholesterol to 30 and 40 mol %, the extent of lipid mixing decreases rapidly.

The overall extent of lipid mixing is higher in PG/Chol LUVs than in PC/Chol LUVs. For the fusion of two LUVs, their merging bilayers must overcome the hydration and electrostatic barriers to attain minimum spatial proximity.^56,57^ It is initiated by the short-range hydrophobic interactions among the merging bilayers but the extent of fusion is mainly dictated by long-range electrostatic interactions among the merging bilayers.^12^ In the case of POPC/Chol LUVs, electrostatic interactions are expected to be high due to their strong net positive charge in the presence of ionic liquids (see Figure S3a), which prevents this close approach and therefore membrane fusion. On the other hand, in the case of POPG/Chol LUVs, the electrostatic interactions are low due to their nearly neutral charge in the presence of ionic liquids (see Figure S3b). This explains the reason behind the lower fusion propensity of POPC/Chol LUVs compared to POPG/Chol LUVs in the presence of ILs.

## Conclusions

In this work, we have evaluated how varying cholesterol levels impact the interaction between 1-dodecyl-3-methylimidazolium bromide ([C_12_MIM]^+^Br^–^) ionic liquid and biomimicking membranes made of either zwitterionic POPC or negatively charged POPG. We observed that both types of LUVs (POPC and POPG) show a reduction in membrane permeability induced by [C_12_MIM]^+^ with increasing cholesterol content. This effect is significantly more pronounced in POPG membranes. In good agreement with this, it was shown that the lipid chain order parameters decrease in the presence of the IL much more in POPC than in POPG membranes. Therefore, one can conclude that [C_12_MIM]^+^ counteracts the lipid condensation effect of cholesterol, especially for POPC membranes. Further, the membrane permeability and order parameter results are also supported by a pressure–area isotherm study, revealing that [C_12_MIM]^+^ decreases the lift-off area of cholesterol-containing POPC and POPG monolayers in a cholesterol-dependent manner. With the increase of the cholesterol content, the lift-off area decreases in both POPG and POPC monolayers; however, this decrease is predominant in the case of POPG monolayers. This reduction in the lift-off area indicates an increase in the order of lipid in the monolayer, correlated with decreased membrane permeability. Also, the membrane fusion of POPC LUVs induced by [C_12_MIM]^+^Br^–^ is notably reduced with increasing cholesterol content in the membrane.

Overall, the effects of [C_12_MIM]^+^Br^–^ on lipid membranes are somewhat attenuated by the presence of cholesterol. Overall, the effect of the IL is more pronounced for POPC compared to POPG membranes, suggesting that electrostatic interactions may influence the membrane interaction of [C_12_MIM]^+^Br^–^. This work holds significant potential for biomedical applications because ILs are being investigated for use in drug delivery systems. The IL interaction with cholesterol can influence the stability and permeability of lipid membranes, ultimately affecting the efficacy of drug delivery through these membranes. Understanding these interactions is critical for optimizing drug delivery strategies and enhancing therapeutic outcomes.

## Supporting Information Available

Fitting of dye leakage profiles of PC/Chol (10:0, 9:1, 8:2, 7:3, 6:4) and PG/Chol (10:0, 9:1, 8:2, 7:3, 6:4) LUVs in the presence of [C_12_MIM]^+^Br^–^ (Figure S1); hydrodynamic diameter of PG LUVs with different cholesterol amount in dependence of the IL concentration (Figure S2), and ζ-potential of PC/Chol and PG/Chol LUVs as a function of [C_12_MIM]^+^Br^–^ concentration (Figure S3) (PDF)
