# Supplementary material for: Role of Cholesterol in Interaction of Ionic Liquids with Model Lipid Membranes and Associated Permeability
Source: J Phys Chem B. 2024 May 25;128(22):5407–18. doi: 10.1021/acs.jpcb.4c01531 (PMC11163423; doi:10.1021/acs.jpcb.4c01531)
Supplement: Supplementary file 1 — jp4c01531_si_001.pdf [file jp4c01531_si_001.pdf]

Supporting Information

Role of Cholesterol in Interaction of Ionic Liquid with Model Lipid Membranes and Associated Permeability

Sandeep Kumar[#a], Navleen Kaur[#a], Prashant Hitaishi[b], Sajal Kumar Ghosh[b], Venus Singh Mithu[#c], Holger A. Scheidt[d*]*

[#]Department of Chemistry, Guru Nanak Dev University, Amritsar 143005, India

Present at: [a]Institute for Bioscience and Biotechnology Research, 9600 Gudelsky Drive, Rockville, Maryland 20850, United States

[b]Department of Physics, School of Natural Sciences, Shiv Nadar Institute of Eminence, NH91, Tehsil Dadri, G. B. Nagar, Uttar Pradesh 201314, India

Present at: [c]Max Planck Institute for Multidisciplinary Sciences, Göttingen 37077, Germany
[d]Institute for Medical Physics and Biophysics, Leipzig University, Leipzig, Germany

AUTHOR INFORMATION

Corresponding Authors:

*HAS: holger.scheidt@medizin.uni-leipzig.de and VSM:vmithu@mpinat.mpg.de

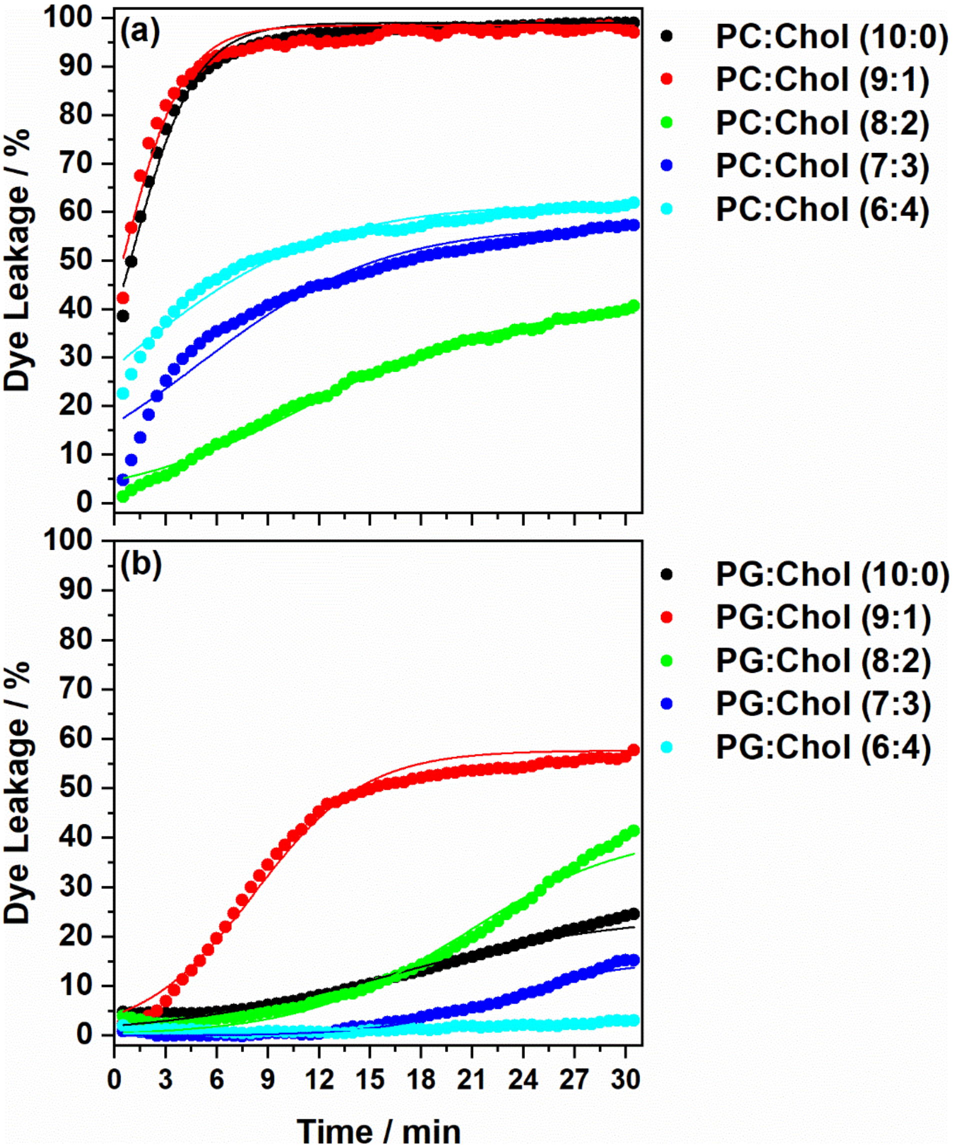

Figure S1. Time-based dye leakage from (a) PC:Chol (10:0, 9:1, 8:2, 7:3, 6:4), and (b) PG:Chol (10:0, 9:1, 8:2, 7:3, 6:4) LUVs upon addition of 0.6 mM $[C_{12}MIM]^{+}Br^{-}$. Solid lines represent sigmoidal fittings. The dye leakage data for PC:Chol (10:0) and PG(10:0) in the presence of $[C_{12}MIM]^{+}Br^{-}$ are adapted from our previous publication.[1]

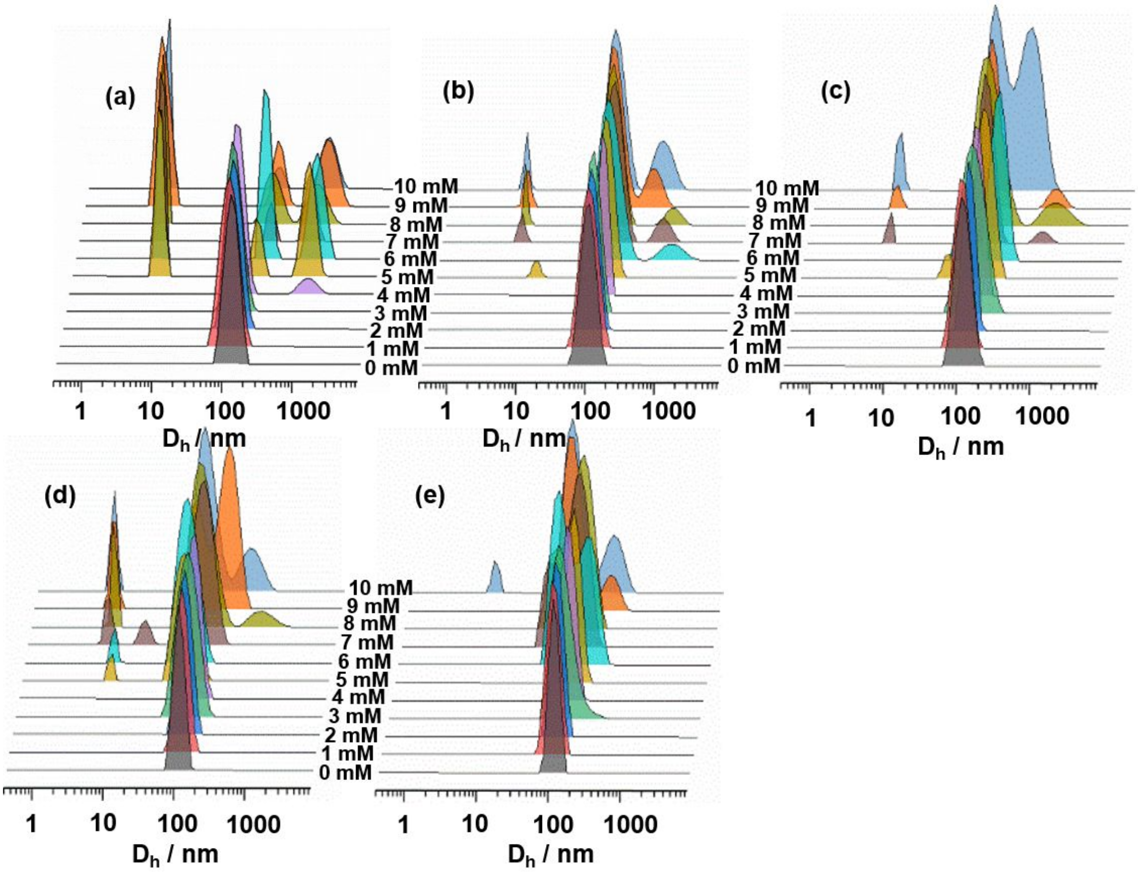

Figure S2. Hydrodynamic diameter (D_h) of (a) PG:Chol (10:0), (b) PG:Chol (9:1), (c) PG:Chol (8:2), (d) PG:Chol (7:3), and (e) PG:Chol (6:4) LUVs at 25 °C after the addition of $[C_{12}MIM]^+Br^-$ at the indicated concentrations. The total phospholipid concentration in the LUVs is 0.275 mM in all cases. The hydrodynamic diameter of PG:Chol (10:0) in the pre- and absence of $[C_{12}MIM]^+Br^-$ are adapted from our previous publication.[2]

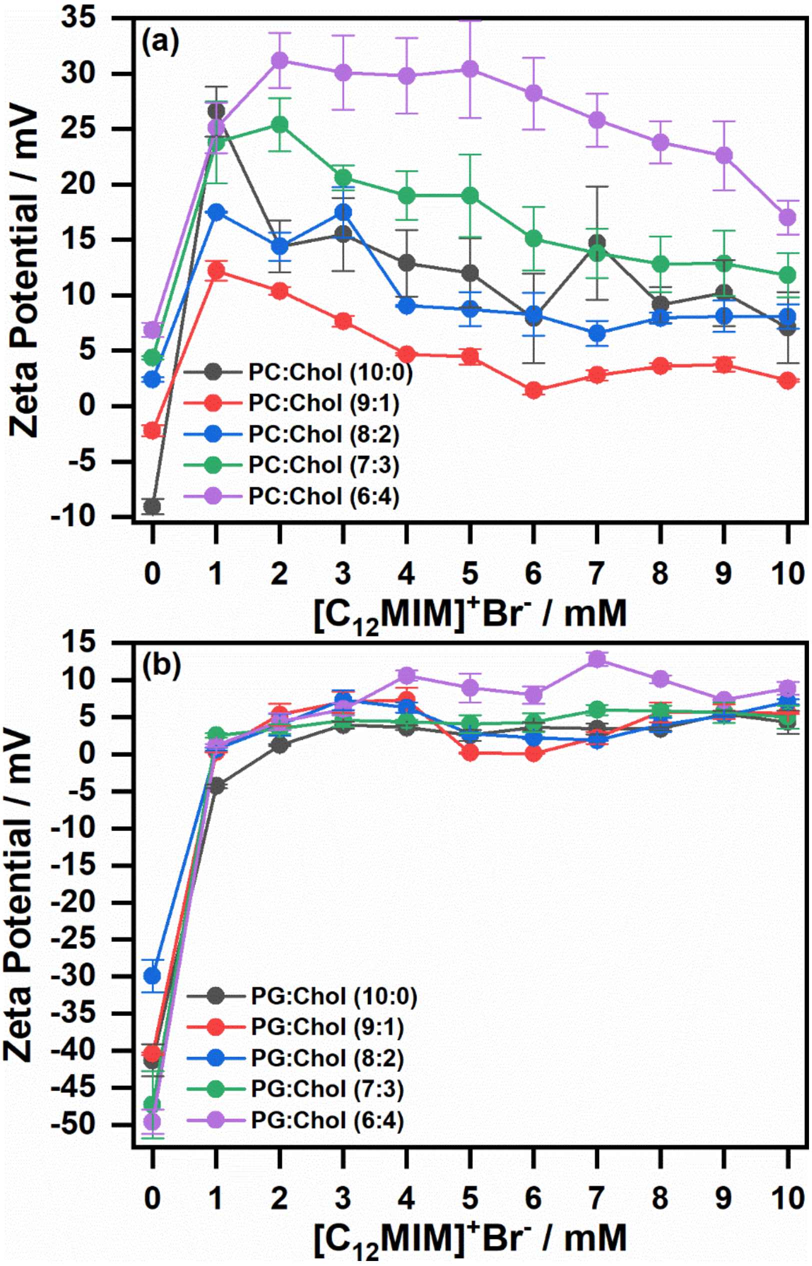

Figure S3. The change in ζ-potential of (a) PC:Chol (10:0, 9:1, 8:2, 7:3, 6:4) and, (b) PG:Chol (10:0, 9:1, 8:2, 7:3, 6:4) LUVs as a function of $[C_{12}MIM]^{+}Br^{-}$ concentration. The change in ζ-

potential of PC:Chol (10:0) and PG:chol (10:0) LUVs in the pre- and absence of $[C_{12}MIM]^+Br^-$ are adapted from our previous publication.[3]

REFERENCES

1. Kumar, S.; Fischer, M.; Kaur, N.; Scheidt, H. A.; Mithu, V. S. Impact of lipid ratio on the permeability of mixed phosphatidylcholine/phosphatidylglycerol membranes in the presence of 1-dodecyl-3-methylimidazolium bromide ionic liquid. *J. Phys. Chem. B* **2021**, *126*, 174-183.

2. Kumar, S.; Kaur, N.; Mithu, V. S. Amphiphilic Ionic Liquid Induced Fusion of Phospholipid Liposomes. *Phys. Chem. Chem. Phys.* **2020**, *22*, 25255-25263.

3. Kumar, S.; Fischer, M.; Kaur, N.; Scheidt, H. A.; Mithu, V. S. Impact of lipid ratio on the permeability of mixed phosphatidylcholine/phosphatidylglycerol membranes in the presence of 1-dodecyl-3-methylimidazolium bromide ionic liquid. *J. Phys. Chem. B* **2021**, *126*, 174-183
